# Tunable Charge Transport Properties Through Precise *π*‐Stacking Modulation in Isostructural Porous Molecular Conductors

**DOI:** 10.1002/anie.202515533

**Published:** 2025-12-31

**Authors:** Liyuan Qu, Hiroaki Iguchi, Kenta Ueno, Shinya Takaishi, Masahiro Yamashita, Chanel F. Leong, Deanna M. D'Alessandro, Takao Tsumuraya, Wakana Matsuda, Shu Seki, Ryotaro Matsuda

**Affiliations:** ^1^ Department of Chemistry and Biotechnology, School of Engineering, and Department of Materials Chemistry Graduate School of Engineering Nagoya University Chikusa‐ku Nagoya 464–8603 Japan; ^2^ Department of Chemistry Graduate School of Science Tohoku University 6‐3 Aramaki‐Aza‐Aoba, Aoba‐ku Sendai Miyagi 980–8578 Japan; ^3^ School of Chemical Science and Engineering Tongji University Shanghai 559 200092 P.R. China; ^4^ School of Chemical & Biomolecular Engineering The University of Sydney New South Wales 2006 Australia; ^5^ Magnesium Research Center Kumamoto University Kumamoto 860–8555 Japan; ^6^ Department of Molecular Engineering Graduate School of Engineering Kyoto University Nishikyo‐ku Kyoto 615–8510 Japan

**Keywords:** Charge mobility, Electronic conductivity, Metal–organic framework, Naphthalenediimide, Redox activity

## Abstract

Understanding the structure–property relationships in electrically conductive metal–organic frameworks (MOFs) is critical for their rational design toward practical applications. Since single crystals of MOFs with through‐space conductive π‐stacked columnar structures are relatively easy to obtain, their structures can be determined with high accuracy. However, elucidating those structure–property relationships without interference from carrier scattering and variations in carrier concentration remains challenging. Herein, we synthesized three isostructural porous molecular conductors (denoted as **PMC‐3**) via electrocrystallization using a redox‐active *N,N’*‐di(4‐pyridyl)‐1,4,5,8‐naphthalenetetracarboxdiimide (NDI‐py) ligand and ZnX_2_ (X = Cl, Br, I). Single crystals of **PMC‐3** exhibit high electrical conductivity (∼10^−3^ S cm^−1^), comparable to the highest values reported for NDI‐based crystalline materials. Moreover, **PMC‐3** serves as a model system for probing structure–property relationships in through‐space conductive MOFs, offering three key advantages. First, the absence of counterions, eliminating carrier scattering; second, identical carrier concentrations across the series, allowing isolation of the effects of *π*‐stacking geometry on transport properties; and third, tunable *π*‐stacking geometries via halide ligand substitution. As a result, a linear correlation between the lattice parameter along the stacking axis and intrinsic charge transport properties is revealed, representing a significant advance in understanding charge transport in through‐space conductive MOFs.

## Graphical abstract

An intrinsic structure–property relationship in through‐space conductive MOFs is investigated using a series of counterion‐free porous molecular conductors (PMC‐3) with carrier‐doped π‐stacked columns. The constant carrier density and halide‐dependent tunable π‐stacking geometry in PMC‐3 enable the discovery of a linear correlation between the lattice parameter along the π‐stacking direction and charge transport properties.

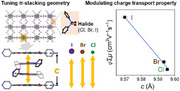

## Introduction

Metal–organic frameworks (MOFs), also known as porous coordination polymers (PCPs), are a class of versatile and tunable hybrid organic–inorganic materials with growing applications in areas such as gas separation,^[^ ^1^ ^]^ chemical sensing,^[^ ^2^ ^]^ and, more recently, electronic devices.^[^ ^3^ ^]^ In particular, MOFs that possess both porosity and electronic conductivity have attracted attention as energy storage materials, such as high‐density capacitors without the need for additional binders or conductive additives.^[^ ^4^ ^]^ Advancing the future applications of electrically conductive MOFs requires a deep understanding of their structure–property relationships, which is key to improving their functionalities. However, the design principles appropriate for investigating such relationships remain scarcely explored.^[^ ^5^, ^6^, ^7^, ^8^, ^9^ ^]^ Since common MOFs are typically insulators due to the combination of hard metals and ligands, resulting in localized frontier orbitals,^[^ ^10^ ^]^ several approaches have been proposed to induce conductivity in MOFs. The through‐bond approach has been employed to promote charge delocalization between metal ions and ligands via coordination bonds, leading to high electronic conductivity and charge mobility.^[^ ^11^, ^12^ ^]^ Over the past decade, two‐dimensional (2D) π‐d conjugated frameworks with stacked layered structures have gained much attention due to their remarkably high conductivity arising from extended charge delocalization. However, the enhancement of conductivity in these systems requires precise energetic alignment and strong orbital overlap between metal ions and ligands, which significantly restricts ligand design to a limited number of systems, such as hexaiminotriphenylene (HITP)^[^ ^13^ ^]^ and benzenehexathiolate (BHT).^[^ ^14^ ^]^ This constraint limits the structural diversity of conductive MOFs; therefore, varying the metal ions has been the most common strategy for studying their structure–property relationships.^[^ ^5^, ^7^, ^8^, ^9^ ^]^ A notable exception is the tuning of interlayer interactions by introducing alkyl chains into the ligand.^[^ ^6^ ^]^ Moreover, except for a few cases,^[^ ^7^, ^9^ ^]^ these highly conductive MOFs are mostly obtained as powders or microcrystals that are unsuitable for single‐crystal X‐ray diffraction (SXRD) analysis, thereby hindering the atomic‐level structural resolution essential for understanding intrinsic physical properties. From this perspective, the through‐space approach, which enables spatial charge transport via a well‐constructed π‐stacking structure, represents a promising alternative for studying structure–property relationships. In this approach, the reversibility of coordination‐bond formation facilitates the growth of single crystals—similar to common MOFs but in contrast to the through‐bond conductive and π‐d conjugated frameworks. Nevertheless, the through‐space approach remains relatively underdeveloped, as it often results in low conductivity due to a lack of charge carriers. Even in cases where conductivity is observed,^[^ ^15^, ^16^, ^17^, ^18^, ^19^, ^20^, ^21^, ^22^ ^]^ detailed explanations, particularly regarding charge carrier concentrations, have rarely been reported.^[^ ^22^ ^]^ So far, little attention has been paid to determining the carrier concentrations of through‐bond and through‐space conductive MOFs, due to the difficulty in their accurate determination. Since electrical conductivity depends on both carrier concentration and carrier mobility—the latter governed by molecular and crystal structures—uncertainties in carrier concentration obscure the intrinsic structure–property relationships. Consequently, synthesizing a series of conductive MOFs with constant carrier concentrations and well‐defined crystal structures has remained a significant challenge.

To introduce charge carriers into the π‐stacked structure in the through‐space approach, we recently proposed porous molecular conductors (PMCs), which incorporate radical species within the π‐stacked ligands of coordination frameworks, functioning as molecular conductor units.^[^ ^23^, ^24^, ^25^, ^26^ ^]^ The study of the first PMC, denoted as PMC‐1,^[^ ^23^ ^]^ assembled from the redox‐active naphthalenediimide (NDI)‐based ligand of *N,N*‐di(4‐pyridyl)‐1,4,5,8‐naphthalenetetracarboxdiimide (NDI‐py) and Cd^2+^ ion with a molecular formula of [Cd(NDI‐py)(OH_2_)_4_](NO_3_)_1.3 ± 0.1_·*n*DMA (DMA = *N,N*‐dimethylacetamide), demonstrated that the generation of NDI^•−^ species via electrochemical reduction was the key to constructing a well‐packed infinite one‐dimensional (1D) columnar structure of NDI cores within a coordination framework. Such attractive interactions between radical‐bearing NDI cores have also been used to induce unique phenomena, such as a molecular rotator^[^ ^27^ ^]^ and crystal–amorphous–crystal transitions in a MOF.^[^ ^28^ ^]^ Importantly, the average charge of the NDI core in PMC‐1, which corresponds to the charge carrier concentration, is determined to be −0.7 ± 0.1 based on the empirical formula. The conductivity reaches to 3.3 × 10^−3^ S cm^−1^, which is one of the highest values reported among MOFs utilizing the through‐space approach.^[^ ^29^, ^30^ ^]^ However, the semiconducting behavior of PMC‐1 is inconsistent with its regular stacking structure and the noninteger average charge of NDI core, likely due to carrier scattering arising from electrostatic interactions with randomly distributed NO_3_^−^ counterions in the void space. This inconsistency was also observed in PMC‐2, in which Rb^+^ counterions were likewise disordered in the pores.^[^ ^24^ ^]^ Therefore, eliminating randomly distributed counterions from the void space, along with the systematic synthesis of frameworks with tunable π‐stacking structures, is essential for uncovering the structure–property relationships of through‐space conductive MOFs.

Herein, we systematically designed a series of new PMCs incorporating different halide ions not only as the charge‐compensating components but also as coordinating ligands so as to suppress counterion incorporation within the void space. Moreover, variations in the bulkiness of halide ions (Cl^− ^< Br^− ^< I^−^) were used as a strategy to tune the stacking geometry of the frameworks. We report three isostructural frameworks, [Zn(OH_2_)_4_(NDI‐py)][ZnX_2_(NDI‐py)]_2_·*n*DMA·*m*H_2_O (X = Cl, Br, I), denoted generally as **PMC‐3**, and specifically as **PMC‐3‐X** (X = Cl, Br, I) when referring to the each halide variant. This series of **PMC‐3** compounds contains an identical number of halide ions, resulting in a constant average charge (−0.67) on the NDI cores across the series. This feature allows us to isolate the effects of π‐stacking geometry on charge‐transport properties through halide substitution. The largest halide ion (I^−^) provides the shortest π‐stacking distance between NDI cores and the superior charge‐transport property, indicating that the bulkiness of halide ions acts as “negative chemical pressure”.^[^ ^31^ ^]^ Such precise tuning of intrinsic charge mobility through control of π‐stacking geometry at constant carrier concentration has not been reported for through‐space conductive MOFs.

## Results and Discussion

### Syntheses and Structures

**Figure 1:**
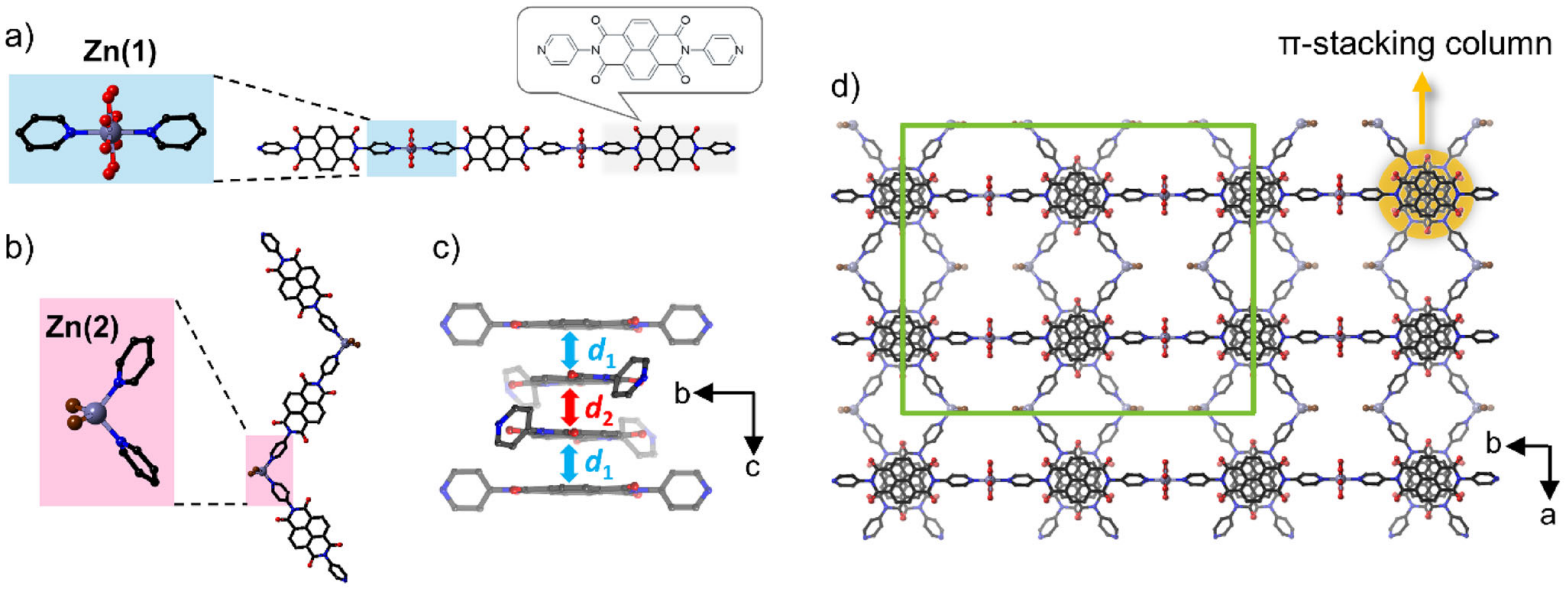
Crystal structure of **PMC‐3‐Br**. a) Linear coordination polymer with the octahedral Zn^2+^(1) coordination center (Inserted box shows the molecular structure of NDI‐py). b) Zigzag coordination polymer with the tetrahedral Zn^2+^(2) coordination center. c) The *π*‐stacked columnar structure formed at NDI cores. d) Perspective view of the packing structure of **PMC‐3‐Br** along the *c* axis. Brown, Br; Gray, Zn; red, O; blue, N; black, C. The color intensity decreases toward the background in c) and d). The green frame shows the unit cell. The H_2_O ligands coordinated to Zn^2+^(1) ions are disordered with half occupancy. Hydrogen atoms are omitted for clarity.

Three isostructural frameworks of **PMC‐3‐Cl**, **PMC‐3‐Br**, and **PMC‐3‐I** were synthesized by applying a constant direct current of 30 µA to a solution of NDI‐py and corresponding ZnX_2_ (X = Cl, Br, I) in DMA at room temperature (RT). Rectangular‐prism‐like black crystals were isolated from the cathode (a Pt–Ir alloy wire) after several days. The SXRD analysis revealed that **PMC‐3** crystallizes in the orthorhombic space group *Cmme* (Table S1).^32^ Figure 1 shows the crystal structure of **PMC‐3‐Br** as a representative example. Two independent Zn centers exist in the asymmetric unit. The Zn^2+^(1) ion adopts an octahedral geometry, coordinated by four H_2_O molecules in the equatorial plane and two NDI‐py ligands at the axial positions, forming infinite 1D linear coordination polymer chains (Figure 1a). The Zn^2+^(2) ion is coordinated by two NDI‐py ligands and two halide ions in tetrahedral geometry, giving rise to infinite 1D zigzag coordination polymer chains (Figure 1b). The linear and zigzag chains interlace in a 1:2 ratio, and thus, the formula of the coordination network is given as [Zn(OH_2_)_4_(NDI‐py)][ZnX_2_(NDI‐py)]_2_. The NDI cores then form 1D infinite π‐stacked columns along the *c* axis, with a rotation of approximately 60° between adjacent cores (Figures 1c and S1), resulting in a porous structure (Figure 1d)

As the residual electron density in the void space was too disordered to be modeled, we performed NMR spectroscopy and elemental analysis to estimate the components in the void space (Figure S2). The single crystals of **PMC‐3** were dissolved in *d*_6_‐DMSO to acquire the ^1^H NMR spectra. As shown in Figure S3, the spectra indicate the presence of approximately eight DMA molecules per [Zn(OH_2_)_4_(NDI‐py)][ZnX_2_(NDI‐py)]_2_ unit. Elemental analyses (C, H, N and halogen) suggest that **PMC‐3‐Cl** and **PMC‐3‐I** contain eight DMA and three H_2_O molecules in the void space per formula unit, consistent with the NMR results. In the case of **PMC‐3‐Br**, only six DMA molecules are estimated based on elemental analysis, likely due to the liberation of three H_2_O and two DMA molecules during the drying process. Thermogravimetric analysis (TGA) (Figure S4) shows that **PMC‐3** begins to lose lattice solvent molecules even at RT. In particular, **PMC‐3‐Br** displayed a faster weight loss than **PMC‐3‐Cl** and **PMC‐3‐I** below 75 °C. This result supports the lower solvent content of **PMC‐3‐Br** suggested by elemental analysis. Based on these results, the empirical formula of **PMC‐3** is estimated to be [Zn(OH_2_)_4_(NDI‐py)][ZnX_2_(NDI‐py)]_2_·8DMA·3H_2_O, revealing that no counterions are present in the void space. Accordingly, the average charge on the NDI cores is clearly calculated to be −0.67, indicating that the **PMC‐3** frameworks contain an identical amount of NDI^•−^ species within the π‐stacked columns. The crystallinity of **PMC‐3** gradually decreases due to the liberation of the lattice solvent molecules upon heating, as observed by powder X‐ray diffraction (PXRD) (Figure S5). Nevertheless, it exhibits CO_2_ adsorption corresponding to approximately 10% of the amount expected from the void volume of the initial crystal structure (Figure S6). This result suggests that some voids remain accessible even after the decrease in crystallinity. In contrast, no N_2_ adsorption is observed at 77 K, likely due to the pore‐blocking effect^[^ ^33^ ^]^ as well as the larger kinetic diameter of N_2_ compared to CO_2_.

Upon closer examination of the isostructural **PMC‐3** frameworks, variations in the lattice parameter *c* and interplanar distances between NDI cores were observed, depending on the halide ion. Here, the interplanar distance between the linear and zigzag chains is defined as *d*_1_, and that between adjacent zigzag chains as *d*_2_ (Figure 1c). The values of *d*_1_ and *d*_2_ were determined by measuring the distances between the centroids of each NDI core (20 atoms except hydrogen). Variations in the lattice parameter *c*, as well as in *d*_1_ and *d*_2_, which range from 3.147–3.220 Å, are summarized in Table 1. These differences among the three **PMC‐3** compounds indicate that the packing structures of the NDI cores can be tuned by altering the halide ions. Combined with the identical average charge on the NDI cores, this structural tunability makes **PMC‐3** a suitable system for investigating the relationship between π‐stacking structure and charge transport properties, free from the influence of variations in carrier concentration.

**Table 1:** Interplanar distances between adjacent NDI cores on linear/zigzag chains (*d*_1_) and zigzag/zigzag chains (*d*_2_), as well as the lattice parameter *c*.

	PMC‐3‐Cl	PMC‐3‐Br	PMC‐3‐I
*d*_1_/Å	3.220	3.214	3.212
*d*_2_/Å	3.155	3.165	3.147
*c*/Å	9.596	9.594	9.570

### Electronic States

**Figure 2:**
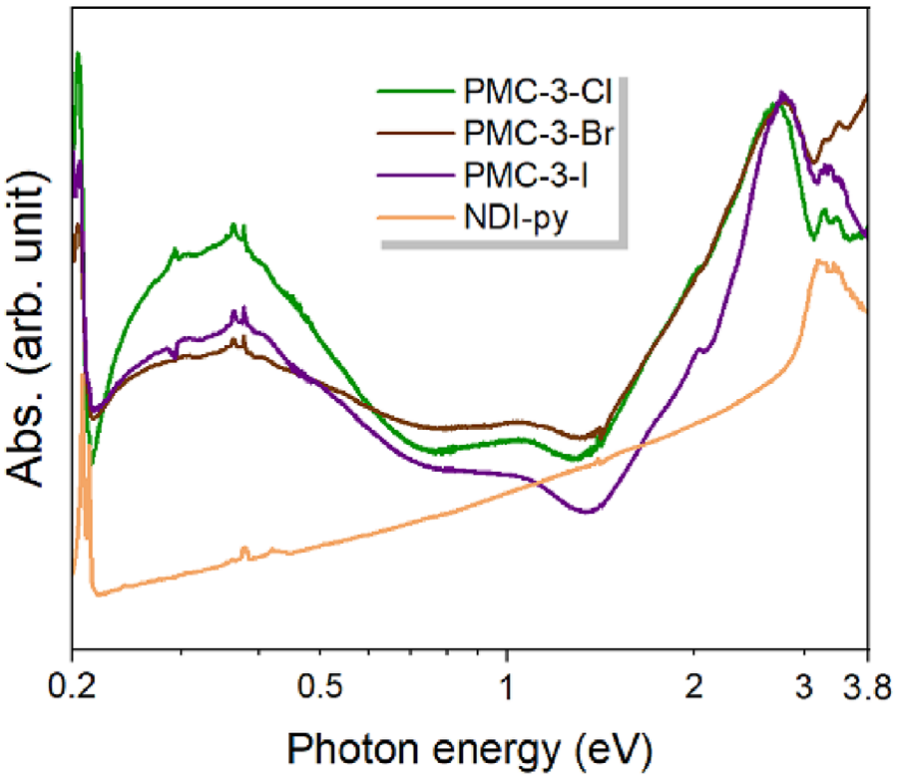
Solid‐state absorption spectra of NDI‐py (orange) dispersed in KBr pellet and **PMC‐3** dispersed in KX pellet (X = Cl (green), Br (brown) and I (purple)), acquired under an N_2_ atmosphere.

To confirm the presence of NDI^•−^ species, solid‐state absorption spectroscopy was performed. The UV–vis–IR absorption spectra of **PMC‐3** dispersed in KX (X = Cl, Br, I) pellets were acquired in a N_2_ atmosphere. As illustrated in Figure 2, an absorption band within 3.0–3.5 eV (354–413 nm), corresponding to the π–π* transition of the neutral NDI (NDI^0^) core, was observed in both neutral ligand NDI‐py and **PMC‐3**, suggesting the presence of NDI^0^ species in **PMC‐3**. In contrast, several absorption bands around 2.8 eV (443 nm) and a small band around 2.0 eV (620 nm) are ascribed to intramolecular transition of NDI radicals, indicating the presence of NDI^•−^ species in **PMC‐3**.^[^ ^34^, ^35^ ^]^ In addition, a small band around 1.0 eV (1,240 nm) and a broad band extending from the near‐IR (NIR) to the IR region (0.25–0.60 eV, 2067–4,960 nm), with a maximum at approximately 0.35 eV, are assigned to through‐space intervalence charge‐transfer (IVCT) bands between two NDI^•−^ cores and between NDI^•–^ and NDI^0^ cores, respectively.^[^ ^27^ ^]^ The O–H stretching mode of water molecules (0.4 eV, 3200 cm^−1^) is also observed. The presence of these IVCT bands supports the π‐stacked columnar structure. Moreover, the appearance of the IVCT band between two NDI^•−^ cores (1 eV) is indicative of the large average charge ranging from −0.5 to −1.0, which is typically observed in π‐stacked molecular conductors.^[^ ^36^ ^]^ Consequently, these results demonstrate that three **PMC‐3** compounds contain both neutral and anionic NDI cores, compatible with their average charge of −0.67.

To gain a better understanding of their electronic states, we conducted the magnetic susceptibility measurement for **PMC‐3‐Cl** and **PMC‐3‐Br** using a superconducting quantum interference device (SQUID) magnetometer. As shown in Figure S7, *χ*_M_ *T* values (*χ*_M_: molar spin susceptibility, *T*: temperature) of **PMC‐3‐Cl** and **PMC‐3‐Br** are roughly constant (0.020–0.027 emu K mol^−1^) from 10 to 120 K and gradually increase above 120 K. The *χ*_M_ *T* values are significantly smaller than the expected value of 2 × 0.375 = 0.75 (emu K mol^−1^), where two radical electrons exist per formula. The effective spin density is estimated to be 2.6%–3.6%, suggesting the spin quenching by dimerization of NDI cores. The *χ*_M_–*T* data are reasonably reproduced by a model consisting of a Curie term and a singlet–triplet thermal excitation component as shown in Figure S7 and fitting equation and parameters described in the Supporting Information. Consequently, the results of magnetic susceptibility are consistent with charge localization at the dimer site. First‐principles band‐structure calculations of **PMC‐3** (Figure S8) clearly show that the bands disperse only along Γ–Z direction, indicating a 1D band structure. The overlap integrals in all other crystallographic directions are extremely small, consistent with the 1D π‐stacked columnar structures spatially isolated by the surrounding pores. The calculated band gaps for three **PMC‐3** compounds are all approximately 0.05 eV, which further supports the charge‐localized model discussed above.

### Electrical Conductivity

**Figure 3:**
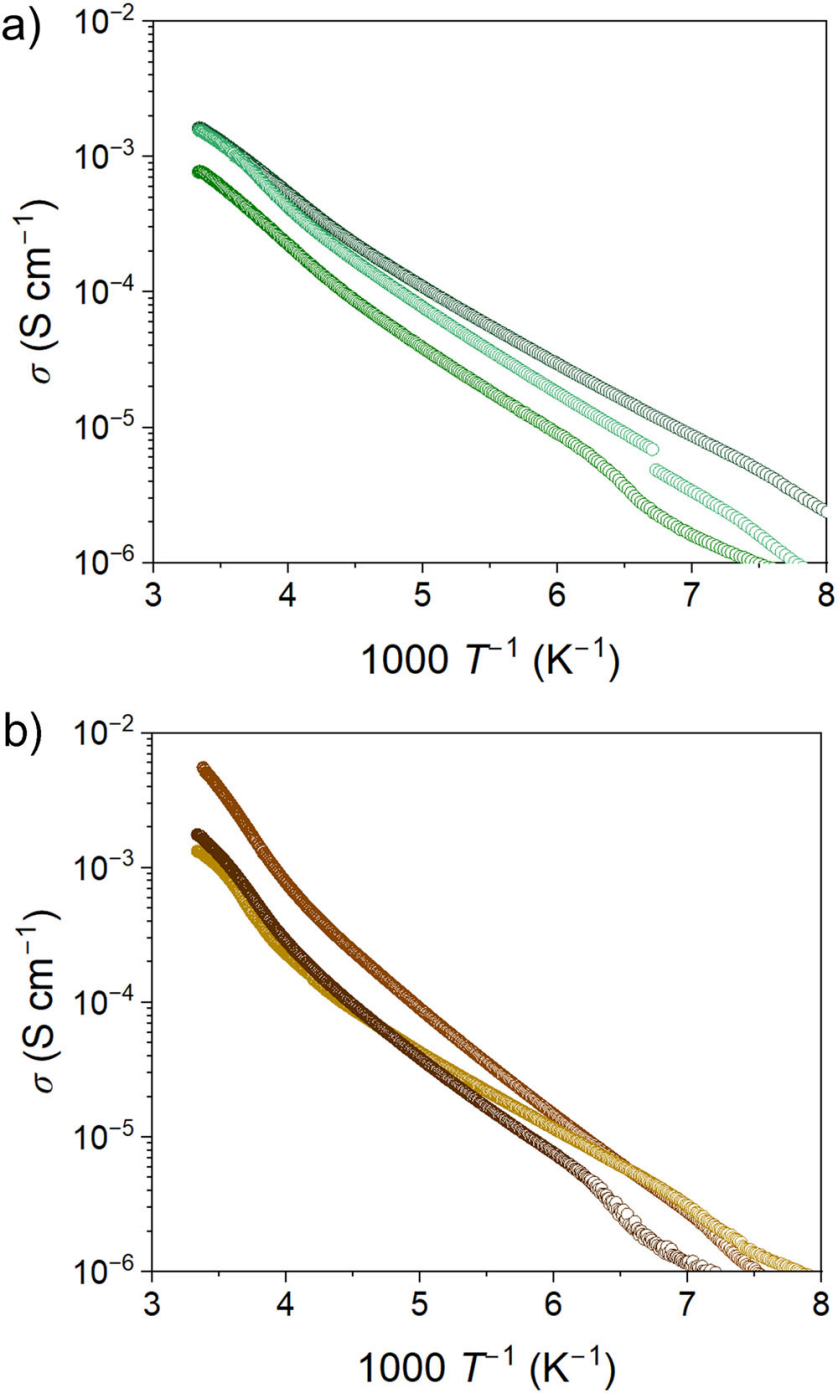
Temperature dependence of electrical conductivity (*σ*) of a) **PMC‐3‐C**l and b) **PMC‐3‐Br** measured in single crystals along the *π*‐stacking direction.

The presence of the IVCT bands and the slightly varied 1D columnar structure among NDI cores encouraged us to investigate the electrical conductivity (*σ*) of **PMC‐3**. Initially, *σ* was measured in single‐crystal form along the π‐stacking direction (crystallographic *c* axis) via a two‐contact probe method. As shown in Figure 3, *σ* increases with increasing temperature, which is indicative of semiconducting behavior. In **PMC‐3**, two slightly different π‐stacking distances (*d*_1_ and *d*_2_) are likely to induce an energy gap in the electronic band structure and give rise to the semiconducting nature of the crystals. The values of *σ* were calculated to be (0.77–1.6) × 10^−3^ S cm^−1^ for **PMC‐3‐Cl** and (1.3–5.5) × 10^−3^ S cm^−1^ for **PMC‐3‐Br** at RT (Tables S3, S4). These values are more than 10^7^ times greater than that of the reported neutral‐NDI‐based MOF,^[^ ^37^ ^]^ which also possesses 1D face‐to‐face, well‐packed NDI columnar structures with short interplanar distances of 3.24 Å. Furthermore, they are comparable to the highest *σ* reported for NDI‐based molecular conductors (7.5 × 10^−3^ S cm^−1^)^[^ ^38^ ^]^ and PMCs (7.6 × 10^−3^ S cm^−1^).^[^ ^26^ ^]^ This result demonstrates that introducing radicals as charge carriers can dramatically enhance the conductivity of MOFs constructed via a through‐space approach. The activation energy (*E*_a_) was calculated to be 125–138 meV for **PMC‐3‐Cl** and 126–170 meV for **PMC‐3‐Br**, respectively (details are provided in Tables S3 and S4). The higher *E*_a_ values and the larger scatter observed for **PMC‐3‐Br** compared with **PMC‐3‐Cl** are likely attributable to the faster liberation of lattice solvent molecules. These *E*_a_ values are larger than those obtained by the first‐principles band calculation (∼50 meV). This discrepancy arises from the fact that the GGA–PBE functional used in the band calculation tends to delocalize charge density and systematically underestimate the band gap.

As described above, the crystallinity of **PMC‐3** decreases with the gradual liberation of lattice solvent molecules at RT. In fact, we found many cracks on the single crystals after the conductivity measurement, as shown in Figure S9. The influence of cracks was serious in the case of **PMC‐3‐I**, which was always obtained as thinner and more fragile crystals than **PMC‐3‐Cl** and **PMC‐3‐Br**, preventing the conductivity measurement in a single‐crystal form. Thus, we then measured the current–voltage (*I*–*V*) characteristics of **PMC‐3** in pressed pellet form, where the influence of cracks is incorporated into the grain boundary resistivity. Additionally, since the pellet is confined within an insulating tube and between the electrodes, the liberation of lattice solvent molecules is also suppressed. The *I*–*V* measurements were carried out on 3 mmφ pressed pellets at RT in a N_2_ atmosphere to avoid air oxidation. Three pellets from different batches were prepared for each **PMC‐3**. The results are plotted in Figure S10, and the values of *σ* are listed in Table S5. As a result, *σ* were calculated to be (2.7–6.1) × 10^−5^ S cm^−1^, (0.98–1.1) × 10^−4^ S cm^−1^, and (1.0–1.2) × 10^−4^ S cm^−1^ for **PMC‐3‐Cl**, **PMC‐3‐Br**, and **PMC‐3‐I**, respectively, showing a slight increase in the order of **PMC‐3‐Cl** < **PMC‐3‐Br** <** PMC‐3‐I**. The variations in σ across different batches are likely attributable to slight difference in grain boundaries and contact resistance. To elucidate how halide ions contribute to *σ*, a detailed investigation of the crystal structure is necessary.

### Halide‐Dependent Variation in *π*‐Stacking Geometry

**Figure 4:**
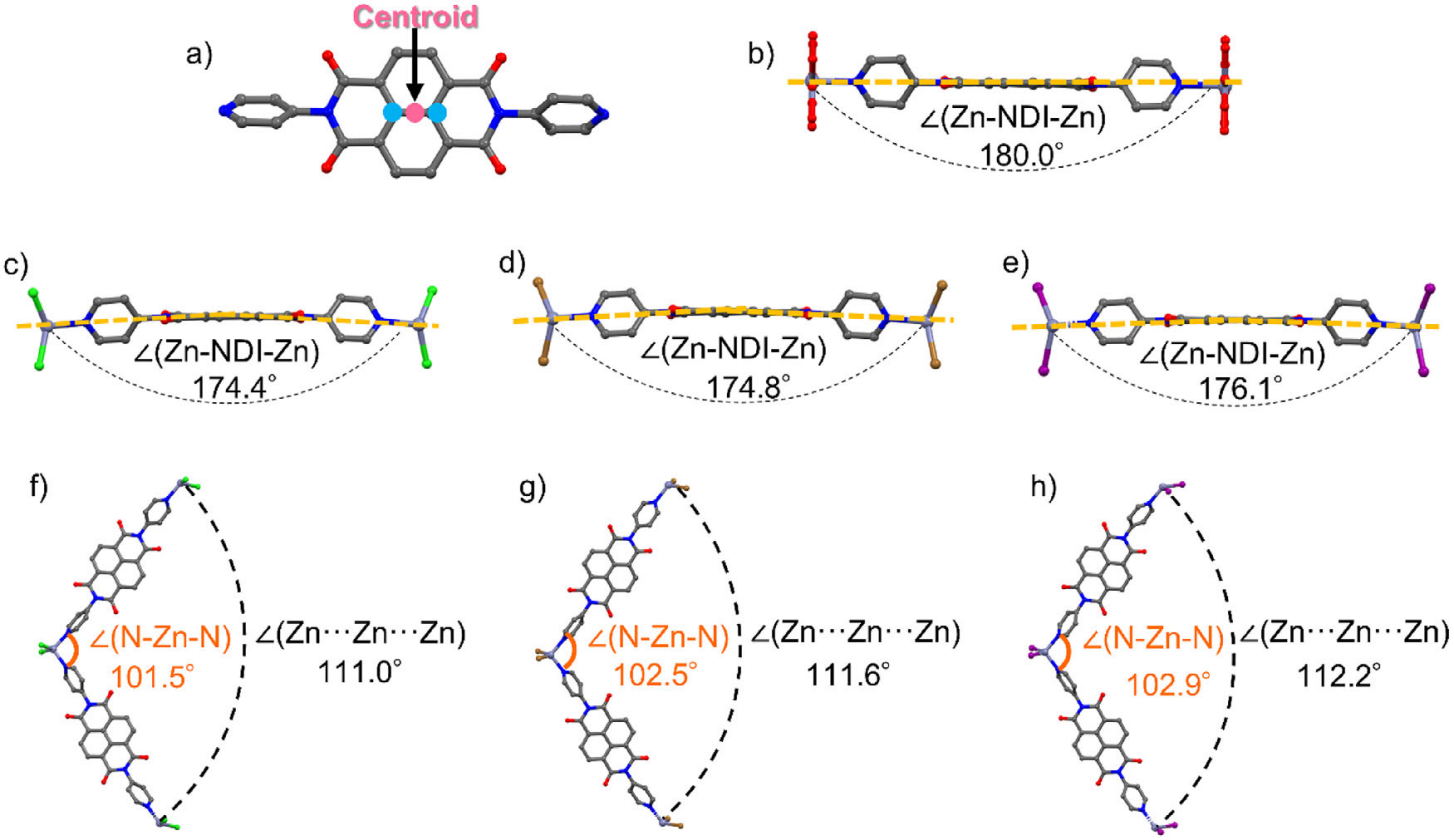
Detailed crystal structures of **PMC‐3**. a) The centroid (shown in pink color) of the two central carbon atoms (shown in blue color) of the NDI core. Visualization of the ∠(Zn–NDI–Zn) extracted from the crystal structure: b) for the linear chain; c)–e) for the zigzag chains in c) **PMC‐3‐Cl**, d) **PMC‐3‐Br**, and e) **PMC‐3‐I**, respectively. Visualization of the ∠(N–Zn–N) and ∠(Zn···Zn···Zn) extracted from the crystal structure of f) **PMC‐3‐Cl**, g) **PMC‐3‐Br**, and h) **PMC‐3‐I**.

The unit cell volume, as well as the lattice parameters *a* and *b* of **PMC‐3**, increase in the order of **PMC‐3‐Cl** < **PMC‐3‐Br** < **PMC‐3‐I**. Considering that the amount of solvent molecules in the void space is the same for the three PMCs, this increase is attributed to the increasing halide ionic radii (cf. ionic radii, Cl^−^: 1.81 Å, Br^−^: 1.96 Å, and I^−^: 2.20 Å).^[^ ^39^ ^]^ On the other hand, the lattice parameter *c* (along the π‐stacking direction) decreases in the order of **PMC‐3‐Cl** > **PMC‐3‐Br** > **PMC‐3‐I**. In other words, the largest radius of I^−^ results in the shortest interplanar distance of NDI cores. To elucidate the origin of this phenomenon, we carefully examined the crystal structures. First, we focused on the planarity of the NDI‐py ligand. Here, the ∠(Zn–NDI–Zn) of each coordination polymer is defined as the angle formed by two Zn^2+^ ions and the centroid of the NDI core, where the centroid is determined as the midpoint between the two central carbon atoms of the NDI ring (Figure 4a). Accordingly, the ∠(Zn–NDI–Zn) in the linear chain is 180° (Figure 4b), while those in the zigzag chain are bent to 174.4°, 174.8°, and 176.1° for **PMC‐3‐Cl**, **PMC‐3‐Br**, and **PMC‐3‐I**, respectively (Figure 4c–e). Because the bending of the NDI‐py ligands suppresses dense π‐stacking along the *c* axis, an increase in the ∠(Zn–NDI–Zn) leads to a flatter NDI‐py ligand and a decrease in the lattice parameter *c*. Next, we consider the reason why the NDI‐py ligands are bent. According to previous reports on NDI‐based crystals,^[^ ^23^, ^24^, ^25^, ^37^, ^40^, ^41^ ^]^ the NDI cores tend to stack with an orientation offset of approximately 60° relative to adjacent units, particularly when radical species are present. This stacking preference is likely due to the enhanced overlap of the LUMO between neighboring NDI cores. To fulfil this orientation offset, the angle of the zigzag chain, defined as the angle formed by three Zn^2+^ ions bridged by two NDI‐py ligands (∠(Zn···Zn···Zn)), should ideally be 120°. However, the actual ∠(Zn···Zn···Zn) values for **PMC‐3‐Cl**, **PMC‐3‐Br**, and **PMC‐3‐I** are 111.0°, 111.6°, and 112.2° (Figure 4f–h), respectively, indicating the presence of structural distortion. Focusing on the tetrahedral coordination geometry of the Zn^2+^ ion, the angles formed between two nitrogen atoms of pyridyl groups and the Zn^2+^ ion (∠(N–Zn–N)) are 101.5°, 102.5°, and 102.9° (Figure 4f–h) for **PMC‐3‐Cl**, **PMC‐3‐Br**, and **PMC‐3‐I**, respectively. The slight increase in the ∠(N–Zn–N) with increasing halide ionic radius is likely due to steric repulsion between the pyridyl rings and the halide ions. As a result, an increase in the halide ionic radius reduces the structural distortion (i.e., the deviation of the actual ∠(Zn···Zn···Zn) from 120°), and thereby enhances the planarity of the NDI‐py ligands in the zigzag chains. This leads to a decrease in the lattice parameter *c*, which behaves like negative chemical pressure.

### Correlation Between the Lattice Parameter *c* and Charge Transport Properties

**Figure 5:**
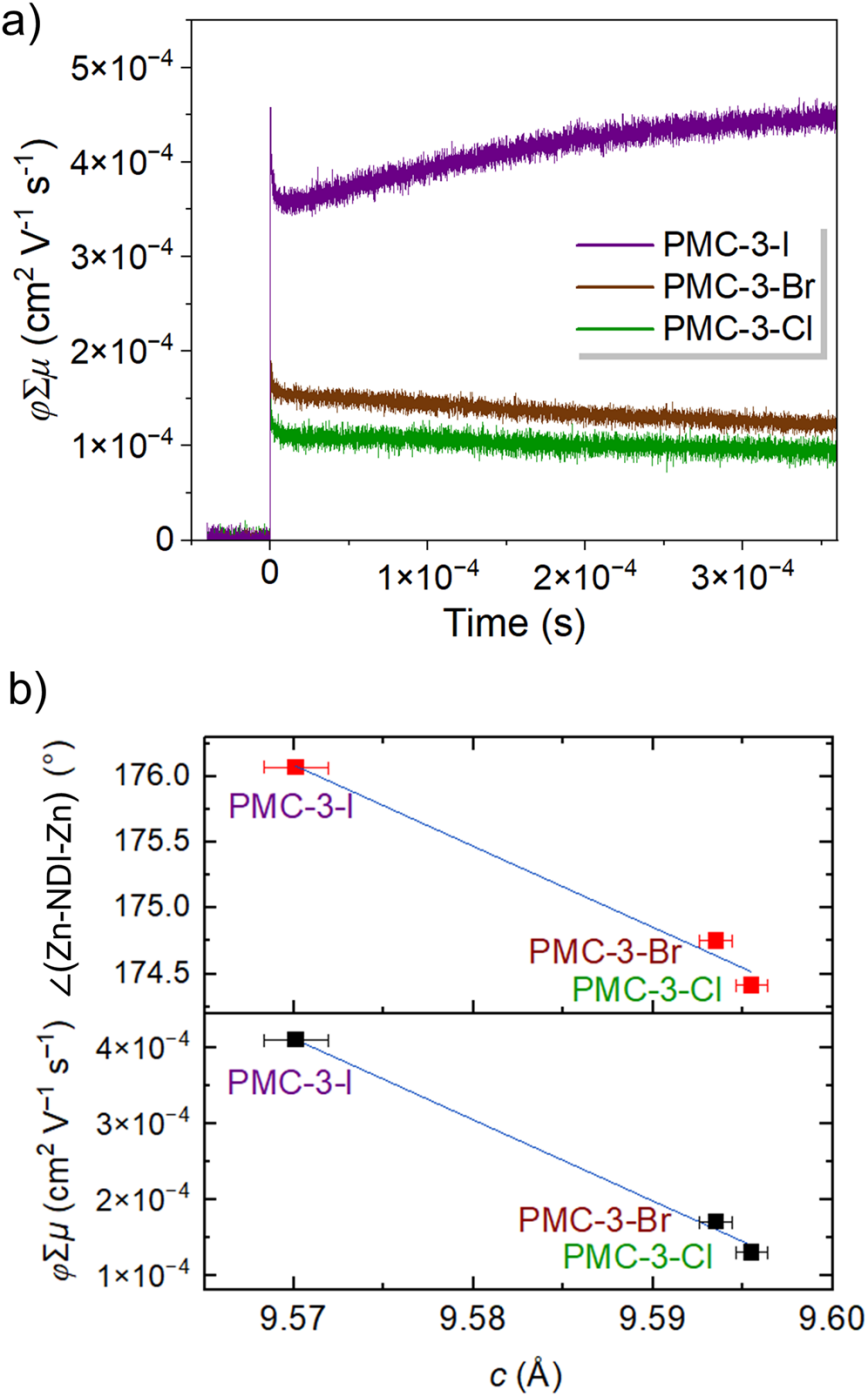
a). Photoconductivity transients of **PMC‐3** upon excitation at 355 nm under an N_2_ atmosphere. b) Dependence of both the ∠(Zn–NDI–Zn) in the zigzag chain and the *φ*Σ*μ* values on the lattice parameter *c* for **PMC‐3‐Cl**, **PMC‐3‐Br**, and **PMC‐3‐I**.

The decrease in the lattice parameter *c* with increasing halide ionic radius corresponds to the trend of increasing *σ* described above. However, the correlation is still unclear because *σ* is affected by extrinsic factors such as grain boundaries and contact resistance. Since the carrier concentration, namely the amount of NDI^•−^ anions, is considered to be identical in **PMC‐3**, the observed variation in *σ* can be ascribed to the difference in carrier mobility, which is expected to correlate with the interplanar distances. Therefore, we conducted flash‐photolysis time‐resolved microwave conductivity (FP‐TRMC) measurements, a powerful technique for probing the intrinsic charge transport properties of materials. FP‐TRMC probes charge transport over a multi‐nanometer length scale in a non‐contact manner, thereby avoiding the influence of grain boundaries, cracks, and contact resistance.^[^ ^42^, ^43^ ^]^ The *φ*Σ*μ* value, where *φ* is the photocarrier generation yield and Σ*μ* represents the sum of the mobilities of both holes and electrons, increased in the order of **PMC‐3‐Cl** < **PMC‐3‐Br** < **PMC‐3‐I** in the powder state, as shown in Figure 5a. The value of *φ*Σ*μ* for **PMC‐3‐I** reached 4.1 × 10^−4^ cm^2^ V^−1^ s^−1^ (Table S6), which is comparable to those of reported NDI‐based organic crystals.^[^ ^42^ ^]^ Furthermore, we illustrated the dependence of both the ∠(Zn–NDI–Zn) of the zigzag chain and the *φ*Σ*μ* values on the lattice parameter *c* in Figure 5b. The larger the ∠(Zn–NDI–Zn) (i.e., the flatter the NDI plane), the shorter the lattice parameter *c*, resulting in a superior *φ*Σ*μ* value. These results support the hypothesis that a shorter interplanar distance facilitates more efficient carrier hopping, thereby leading to higher electrical conductivity. This linear correlation between the lattice parameter *c* and the *φ*Σ*μ* values provides initial evidence for an intrinsic structure–property relationship in through‐space conductive MOFs.

### Redox Activity

Since the series of **PMC‐3** compounds contains the same amount of neutral NDI cores, **PMC‐3‐Br** was selected as a representative compound for the investigation of redox properties by acquiring a solid‐state cyclic voltammogram in 0.1 M LiClO_4_/CH_3_CN at a scan speed of 100 mV s^−1^ (Figure S11a). The voltammogram shows a pair of quasi‐reversible peaks observed at −1.22 and −0.96 V (versus Fc/Fc^+^) in the reduction and oxidation processes, respectively, corresponding to the NDI^0^/NDI^•−^ redox couple.^[^ ^44^ ^]^ In the reduction process, the neutral NDI cores are reduced to NDI^•−^ anions, and in the oxidation process, only these NDI^•−^ anions generated by the former reduction step are likely to be oxidized back to the neutral state, as suggested by the unchanged voltammogram over repeated cycles. The inactivity toward further oxidation from the initial reduction state (average charge of −0.67) suggests its exceptional stability. In addition, similar quasi‐reversible cyclic voltammograms were obtained using electrolyte solutions containing Na^+^, K^+^, and Mg^2+^ ions (Figure S11b–d). These results indicate that various cations can be inserted into the pores of **PMC‐3‐Br**, demonstrating its potential as an electrode material. The cyclic voltammogram of solid NDI‐py in 0.1 M LiPF_6_/CH_3_CN at a scan speed of 100 mV s^−1^ (Figure S12) exhibits a more negative reduction potential (−1.27 V (versus Fc/Fc^+^)) and a larger separation between the reduction and the coupled oxidation peak potentials (ca. 0.5 V). These features indicate that the insertion of Li^+^ ion from the electrolyte solution into the solid is hindered by the absence of pores that can accommodate Li⁺ ions.

**Figure 6:**
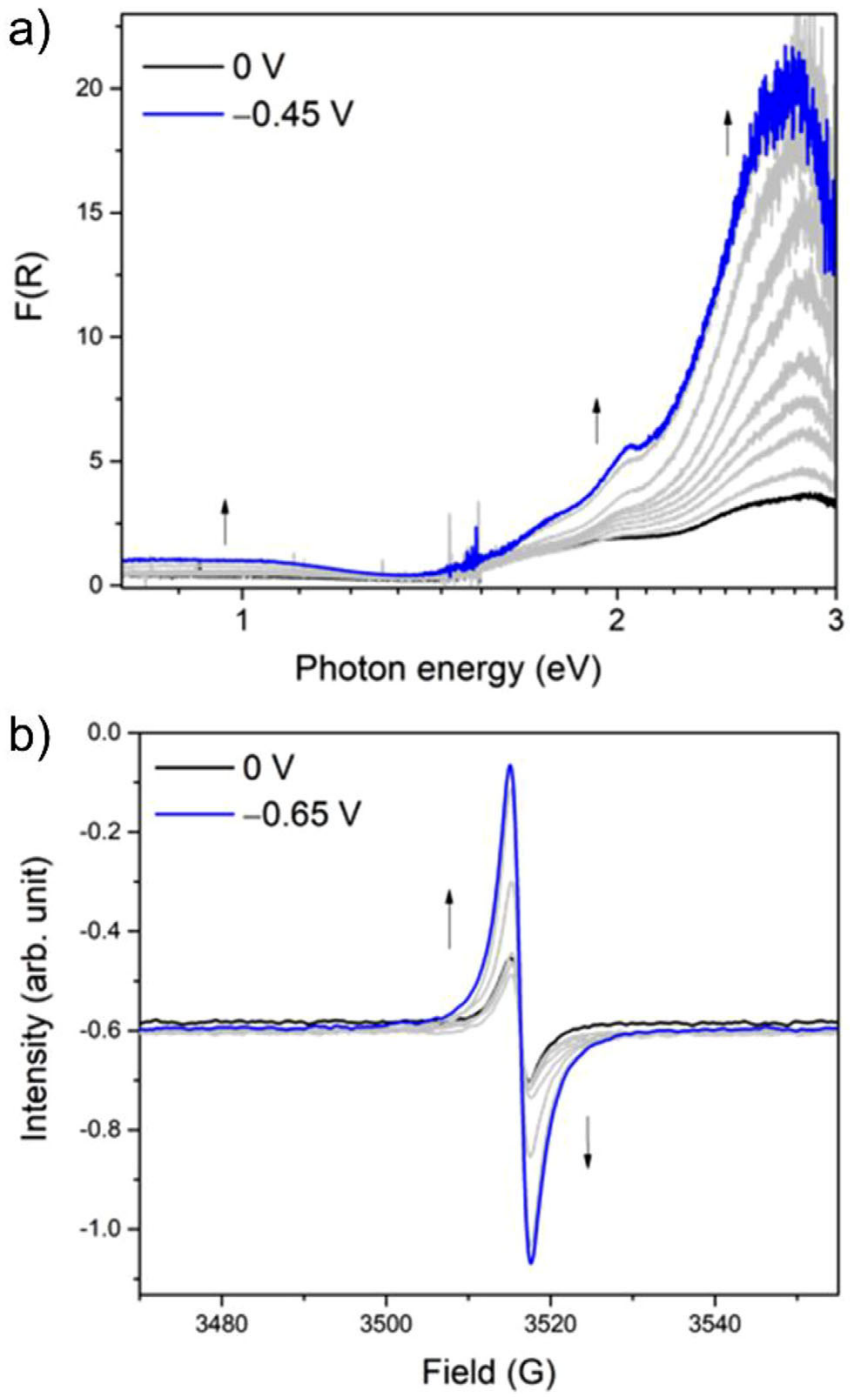
a). Solid‐state spectroelectrochemistry (SEC) in diffuse reflectance mode, F(R), of **PMC‐3‐Br** in 0.1 M LiClO_4_/CH_3_CN upon reduction, showing the increasing intensity of both the absorption bands for intramolecular transition of NDI^•−^ and for IVCT between two NDI^•−^ species, indicating an increasing amount of NDI^•−^ species. b) Solid‐state EPR SEC of **PMC‐3‐Br** in 0.1 M LiClO_4_/CH_3_CN, showing the increasing intensity of the NDI^•−^ signal upon reduction; gray lines show the spectral transition.

To gain further insight into the electronic states of NDI cores in the solid‐state redox process, a combination of electrochemistry and spectroscopies, namely the spectroelectrochemical (SEC) technique was adopted.^[^ ^45^ ^]^ First, solid‐state Vis‐NIR SEC was conducted for **PMC‐3‐Br** under conditions similar to those of the cyclic voltammetry. As shown in Figure 6a, both absorption bands corresponding to the intramolecular transitions of NDI^•−^ species (at 2.0 and 2.8 eV) and the through‐space IVCT between two NDI^•−^ species (around 1.0 eV) were observed prior to potential application (*E* = 0 V), which is in good agreement with the UV–vis absorption spectrum shown in Figure 2. When a reductive potential was applied to the sample, the intensity of these bands gradually increased and finally saturated at a potential of −0.45 V (versus Ag/Ag^+^). The enhancement of both absorption bands indicates an increasing amount of NDI^•−^ species in **PMC‐3‐Br**. In the reverse oxidative process, the intensities of both bands decreased and ultimately returned to the initial state at 0 V, consistent with the reversible cyclic voltammogram. To further demonstrate the increase in NDI^•−^ species during the reduction process, solid‐state electron paramagnetic resonance (EPR) SEC was carried out on **PMC‐3‐Br**. The spectra were acquired in a solution of 0.1 M LiBF_4_/CH_3_CN using a platinum wire as the working electrode. As shown in Figure 6b, a sharp signal (black line) was observed prior to potential application (*E* = 0 V), corresponding to the initially present NDI^•−^ anions in **PMC‐3‐Br**. Upon applying a negative potential up to −0.65 V to the sample, the signal gradually increased in intensity, owing to the increasing amount of NDI^•−^ species during the reduction process. The spectrum nearly returned to its initial state upon reversing the potential back to 0 V (Figures S13, S14). As a result, the electrochemical studies demonstrated that Li^+^ ions can reversibly enter the void space of **PMC‐3‐Br** upon reduction, indicating that the counterion‐free voids in **PMC‐3** provide sufficient space for the accommodation of guest ions. This feature is promising for the development of conductive MOFs with tunable electronic states and properties via postsynthetic electrochemical ion insertion.

## Conclusion

This work presents a pioneering approach for uncovering the structure–property relationships in through‐space conductive MOFs. While carrier doping is a common strategy to enhance their conductivity, it introduces a variable number of counterions into the pores, which often affects carrier transport and obscures the intrinsic structure–property relationships. To overcome this limitation, we prepared three isostructural porous molecular conductors, **PMC‐3‐X** (X = Cl, Br, I), composed of linear and zigzag coordination polymers in a 1:2 ratio. These polymers assemble into 1D infinite π‐stacked columns of NDI cores, which serve as efficient charge transport pathways. This series of **PMC‐3** compounds contains an identical number of halide ions as coordinating ligands, resulting in a constant carrier density (0.67 e^−^ per NDI core) and the absence of counterions within the pores. This feature makes them a suitable model system for investigating intrinsic structure–property relationships in through‐space conductive MOFs. Moreover, the π‐stacking geometry can be tuned by varying the halide ligand, owing to differences in steric repulsion at the ZnN_2_X_2_ (X = Cl, Br, I) tetrahedral coordination site. As the halide ionic radius increases in the order of Cl^−^ < Br^−^ < I^−^, the lattice parameter *c* along the π‐stacking direction decreases, likely reflecting a negative chemical pressure effect. As a result, a linear correlation between the lattice parameter *c* and the *φ*Σ*μ* values, which are related to carrier mobility, has been revealed, providing the first evidence of an intrinsic structure–property relationship in through‐space conductive MOFs. Along with the high electrical conductivity (∼10^−3^ S cm^−1^) and the redox activity of **PMC‐3**, this linear correlation serves as a valuable guideline for designing highly conductive MOFs with through‐space charge transport pathways. In addition, the counterion‐free pores offer the advantage of maximizing the available pore volume. These findings hold great promise for developing conductive MOFs, with future applications as electrode materials in sensors, batteries, and electrocatalysis.

## Supporting Information

The data supporting this article have been included as part of the Supporting Information. The authors have cited additional references within the Supporting Information.^[^ ^46^, ^47^, ^48^, ^49^, ^50^, ^51^, ^52^, ^53^, ^54^, ^55^ ^]^

## Conflict of Interests

The authors declare no conflict of interest.

## Supporting information



Supporting Information

Supporting Information

## Data Availability

The data that support the findings of this study are available from the corresponding author upon reasonable request.
