# Supplementary material for: Tunable Charge Transport Properties Through Precise π‐Stacking Modulation in Isostructural Porous Molecular Conductors
Source: Angew Chem Int Ed Engl. 2025 Dec 31;65(7):e15533. doi: 10.1002/anie.202515533 (PMC12887638; doi:10.1002/anie.202515533)
Supplement: Supplementary file 1 — Supporting Information [file ANIE-65-e15533-s001.pdf]

Supporting Information

Tunable Charge Transport Properties through Precise π-Stacking Modulation in Isostructural Porous Molecular Conductors

Liyuan Qu,*[a] Hiroaki Iguchi,*[a] Ueno Kenta,[b] Shinya Takaishi,[b] Masahiro Yamashita,[b,c] Chanel F. Leong,[d] Deanna M. D'Alessandro,[d] Takao Tsumuraya,[e] Wakana Matsuda,[f] Shu Seki,[f] and Ryotaro Matsuda*[a]

a. Department of Materials Chemistry, Graduate school of Engineering, Nagoya University, Chikusa-ku, Nagoya 464-8603, Japan.
b. Department of Chemistry, Graduate School of Science, Tohoku University, 6-3 Aramaki-Aza-Aoba, Aoba-ku, Sendai, Miyagi 980-8578, Japan.
c. School of Chemical Science and Engineering, Tongji University, Shanghai 559 200092, P.R. China
d. School of Chemical & Biomolecular Engineering, The University of Sydney, Sydney, New South Wales 2006, Australia
e. Magnesium Research Center, Kumamoto University, Kumamoto 860-8555, Japan.
f. Department of Molecular Engineering, Graduate School of Engineering, Kyoto University, Nishikyo-ku, Kyoto 615-8510, Japan.

Table of Contents

Experimental details
Crystallographic data of **PMC-3** (Table S1)
Stacking structure of **PMC-3-Br** (Figure S1)
Voids accommodating lattice solvent molecules in **PMC-3** (Figure S2)
^{1}H NMR spectra (Figure S3)
Thermogravimetry analysis (TGA) of **PMC-3** (Figure S4)
PXRD patterns of **PMC-3-Br** after heating (Figure S5)
Gas sorption isotherm (Figure S6)
Magnetic susceptibility (Figure S7)
Parameters used in fitting χ(Table S2)
Electronic band calculation (Figure S8)
Electrical conductivities of single crystals (Table S3-4)
Photographs of crystals as synthesized and after conductivity measurement (Figure S9)
Electrical conductivities of pressed pellets (Figure S10, Table S5)
Calculated $\phi\Sigma\mu$ of **PMC-3** from FP-TRMC measurement (Table S6)
Solid-state cyclic voltammogram of **PMC-3-Br** (Figure S11)
Solid-state cyclic voltammogram of NDI-py (Figure S12)
Solid-state UV-vis spectroelectrochemistry (SEC) of **PMC-3-Br** (Figure S13)
Solid-state EPR spectroelectrochemistry (SEC) of **PMC-3-Br** (Figure S14)
Reference

Experimental details

All chemicals were used without further purification. The ligand *N,N'*-di(4-pyridyl)-1,4,5,8-naphthalenetetracarboxdiimide (NDI-py) was synthesized according to previously reported procedure.[1] Electrocrystallization was carried out by using direct current (DC) multisources YAZAWA CS-12Z and 0.3 mmϕ electrodes made from platinum-iridium alloy wires with a ratio of 80:20 in an ambient atmosphere. Solid-state UV-Vis-NIR and IR spectra were collected on a Shimadzu UV-3100 and JASCO FT/IR 4200 Fourier Transform Infrared Spectrometer, respectively, by using KCl, KBr and KI dispersed pellets. The pellets were prepared in a glovebox (MBRAUN UNILAB1200/780) filled with Ar gas, and then sealed in a custom cell for IR and UV-Vis-NIR measurement under an inert atmosphere. ^{1}H NMR measurements were performed on a Bruker AV500 at room temperature (RT). Thermogravimetry (TG) was measured on a SHIMADZU DTG-60/60H at a heating rate of 5 °C/min under a constant nitrogen flow (0.1 L/min). The powder X-ray diffraction (PXRD) patterns were acquired on a Bruker D2 PHASER with Cu Kα radiation (λ = 1.5406 Å) at RT in open air. The adsorption isotherm measurements for N_2 and CO_2 were performed using an automatic volumetric adsorption apparatus (BELSORP-max equipped with a cryostatic temperature controller; MicrotracBEL Corp.). The static magnetic susceptibility was measured on polycrystalline sample in the temperature range of 2.0–300 K using a superconducting quantum interference device (SQUID) magnetometer. The calculation of the intrinsic diamagnetic correction was conducted using common Pascal's constants. The *g*-values of the compounds were estimated from their EPR spectra.

Synthesis of [Zn(OH$_2$)$_4$(NDI-py)][ZnCl$_2$(NDI-py)]$_2$·8DMA·3H$_2$O (PMC-3-Cl)

NDI-py (20 mg, 0.0476 mmol) and $ZnCl_2$ (80 mg, 0.59 mmol) were dissolved into 5 mL *N,N*-dimethylacetamide (DMA) with adding 30 μL of pure water. Then, a constant current of 30 μA was applied to the solution. After two days, dark brown rod-like crystals were isolated from the cathode, and subsequently washed by DMA and ethanol followed by drying in an N_2 atmosphere. Elemental analysis calcd (%) for $C_{104}H_{122}Cl_4N_{20}O_{27}Zn_3$: C 51.57, H 5.08, N 11.57, Cl 5.85; found: C 51.28, H 5.28, N 11.56, Cl 5.91.

Synthesis of [Zn(OH$_2$)$_4$(NDI-py)][ZnCl$_2$(NDI-py)]$_2$·8DMA·3H$_2$O (PMC-3-Br)

NDI-py (20 mg, 0.0476 mmol) and $ZnBr_2$ (130 mg, 0.58 mmol) were dissolved into 5 mL *N,N*-dimethylacetamide (DMA) with adding 30 μL of pure water. Then, a constant current of 30 μA was applied to the solution. After two days, dark brown rod-like crystals were isolated from the cathode, and subsequently washed by DMA and ethanol followed by drying in an N_2 atmosphere. This complex tends to rapidly lose some solvent molecules, and the actual chemical formula of the sample used for elemental analysis was [Zn(OH$_2$)$_4$(NDI-py)][ZnBr$_2$(NDI-py)]$_2$·6DMA. Elemental analysis calcd (%) for $C_{96}H_{98}Br_4N_{18}O_{22}Zn_3$: C 48.62, H 4.16, N 10.63, Br 13.48; found: C 48.56, H 3.86, N 10.41, Br 13.74.

Synthesis of [Zn(OH$_2$)$_4$(NDI-py)][ZnI$_2$(NDI-py)]$_2$·8DMA·3H$_2$O (PMC-3-I)

NDI-py (20 mg, 0.0476 mmol) and ZnI_2 (190 mg, 0.60 mmol) were dissolved into 5 mL *N,N*-dimethylacetamide (DMA) with adding 30 μL of pure water. Then, a constant current of 30 μA was applied to the solution. After two days, dark brown rod-like crystals were isolated from the cathode,

and subsequently washed by DMA and ethanol followed by drying in an N_2 atmosphere. Elemental analysis calcd (%) for $C_{104}H_{122}I_4N_{20}O_{27}Zn_3$: C 44.80, H 4.41, N 10.05, I 18.21; found: C 44.48, H 4.58, N 10.10, I 18.27.

Single crystal X-ray structure determination

The diffraction data were collected on two diffractometers: a RIGAKU XtaLab AFC10 diffractometer with a HyPix-6000HE hybrid pixel array detector and a RIGAKU XtaLab P200 diffractometer with a PILATUS3 R 200K-A hybrid pixel array detector. Both are equipped with VariMax Mo Optic with Mo Kα radiation (λ = 0.71073 Å) and a cryogenic equipment. The temperature was set by blowing the chilled nitrogen flow. The structure was solved using direct methods (SHELXT[2]) and followed by Fourier synthesis. Structure refinement was performed using full matrix least-squares procedures with SHELXL[3] on F^2 in the Olex2-1.5.[4] The SQUEEZE method was applied to remove the electron density, which was too dispersed to establish a meaningful molecular structure in the pores.[5]

Electrical conductivity measurement

Variable-temperature conductivity data were collected in a liquid He cryostat of a Quantum Design Physical Property Measuring System (PPMS) MODEL 6000 by using the two-probe method in direct current (DC) mode with Keithley sourcemeter model 2611. The single crystals were attached onto the sample pack using gold wires (15 μmϕ) and carbon paste (Dotite XC-12 in diethyl succinate) along the π-stacking direction (crystallographic *c* axis). The measurements were conducted in a He atmosphere with a cooling/heating rate of 2 K/min.

Electrical conductivities of pressed pellet samples were determined through the *I*–*V* measurements on Keithley sourcemeter model 2450 in a N_2-purged glovebox. The pellets were prepared by sandwiching the crystalline samples into a stainless pellet die assembly equipped with an insulating alumina spacer (3 mmϕ), and then pressed by a Specac Mini-Pellet Press. The thickness of the samples was measured by an electronic disc micrometer. *I*–*V* profiles were collected by connecting the pellet die assembly to the source meter, where the pellet die acts as the electrode.

Solid-state cyclic voltammetry

The solid-state cyclic voltammetry measurement was carried out using a standard three-electrode cell with an ALS/CH Instruments Electrochemical Analyzer Model 620D. The working electrode was a glassy carbon (GC) electrode. Solid was fixed to the surface of the GC electrode by the mechanical attachment method. Platinum and silver wires were used for counter and quasi-reference electrodes, respectively. The potential of the quasi-reference electrode was calibrated by using ferrocene (Fc) as an external standard. The cyclic voltammogram of **PMC-3-Br** was recorded in 0.1 M $LiClO_4$ in dry acetonitrile under a nitrogen flow at a scan rate of 100 mVs^{-1}.

Solid-state NIR-Vis spectroelectrochemical (SEC) measurement

SEC measurements were conducted using a CARY5000 UV/Vis/NIR spectrophotometer equipped with a Harrick Omni Diff probe attachment spanning a range of 400 nm to 2000 nm. This electrochemical setup involved an indium tin oxide (ITO) coated glass plate as working electrode, a Pt wire as counter electrode and a silver wire as quasi-reference electrode in an oxygen free Teflon cell, as described in detail previously.[6] Diffuse reflectance spectra are reported as the Kubelka-

Munk transform, where F(R) = $(1-R)^2/2R$ (R is the diffuse reflectance of the sample as compared to $BaSO_4$).

Solid-state electron paramagnetic resonance (EPR) SEC

EPR SEC data was collected at RT in the X-band using a Bruker EMXnano EPR spectrometer.[7] The one compartment EPR SEC cell was constructed using a Pasteur pipette flame sealed at the thin end and filled to halfway with 0.1 M $LiBF_4$/MeCN as electrolyte. Three electrodes, including a bare Pt counter electrode, a Teflon coated silver wire quasi reference electrode and a Teflon coated Pt working electrode, were connected to separate copper inserts wrapped in Teflon tape and then inserted into the electrolyte to avoid short circuiting. Solid state sample was wrapped into a Pt mesh, then connected to the Pt working electrode. The applied potential was controlled by an eDAQ e-corder 410 potentiostat.

Flash-photolysis time-resolved microwave conductivity (FP-TRMC) measurement

Charge carrier mobilities were investigated by FP-TRMC measurement at room temperature in N_2 atmosphere. The samples were prepared by drop-cast method with polymethylmethacrylate (PMMA) as a binder on quartz substrates. Charge carriers were injected into the materials upon photo-ionization with a third harmonic generation (λ = 355 nm) of a Spectra Physics model INDI-HG Nd: YAG laser pulses at 10 Hz with a pulse duration of ca. 5 ns. The photo density of a 355 nm pulse was modulated from 9.1×10^{15} photons cm^{-2} $pulse^{-1}$. The frequency and power of the probe microwave were set at around 9.1 GHz and 10 mW, respectively, so that the electric field of the microwave was sufficiently small and does not perturb the motion of charge carries. The observed value of photoconductivity converted to the product of the quantum yield ϕ and the sum of charge-carrier mobilities $\Sigma\mu$ by $\phi\Sigma\mu = \Delta\sigma/eI_0F_{light}$, where, e, I_0, F_{light}, and $\Delta\sigma$ are elementary charge, incident photon density of excitation laser (photons per m^2), a correction factor (m^{-1}) and a transient photoconductivity, respectively. The samples were set at the point of electric field maximum in a resonant cavity.

^{1}H NMR spectra

Single crystals of **PMC-3** were dissolved into d_6-DMSO and ^{1}H NMR spectra were acquired. The number of DMA molecules in each **PMC-3** compound was determined as below.

In **PMC-3-Cl**, the ratio of NDI-py and DMA was calculated to be 1:2.67, thus, for the formula of $[Zn(OH_2)_4(NDI\text{-}py)][ZnCl_2(NDI\text{-}py)]_2$, the number of DMA molecules is determined to be 8, which is consistent with the elemental analysis. For the same consideration, the amount of DMA molecules is determined to be 7.2 and 8 for $[Zn(OH_2)_4(NDI\text{-}py)][ZnX_2(NDI\text{-}py)]_2$ (X = Br and I, respectively). Note that the amount of DMA molecules obtained from **PMC-3-Br** is smaller than 8 because of the liberation of solvent molecules during drying. This is compatible with the elemental analysis and the thermogravimetry analysis (TGA) in Figure S4.

Computational Methods

First-principles calculations were performed using the projector augmented-wave (PAW) formalism along with the pseudopotentials implemented in the Vienna *Ab initio* Simulation Package (VASP).[8–10]

The exchange–correlation interactions were described using the Perdew–Burke–Ernzerhof (PBE)

functional within the generalized gradient approximation (GGA).[11] An energy cutoff of 500 eV was used for the plane-wave basis set. For the structural model, structural optimization was performed by fixing the experimentally determined lattice parameters and relaxing only the internal atomic coordinates until the forces on all atoms were below 0.01 eV Å^{-1}. A k-point mesh of 2×2×5 was used for Brillouin-zone sampling.

Because the positions of the hydrogen atoms, particularly those in the H_2O molecules, could not be fully determined from the X-ray diffraction data, a calculation model was constructed in which the hydrogen atoms were placed so as to introduce the least possible symmetry breaking, thereby retaining the maximal number of symmetry operations. The introduction of hydrogen atoms reduces the symmetry from the experimentally determined base-centered orthorhombic *Cmme* (No. 65) structure, and the resulting model can be described within the *C*2/*m* space group (No. 12, base-centered monoclinic). The internal atomic coordinates were then relaxed while preserving the *C*2/*m* symmetry constraints. Because these crystal structures are C-base-centered, we employed the corresponding primitive unit cell containing half the number of atoms of the conventional cell for the band structure calculations.

Table S1 Crystallographic details for **PMC-3** with SQUEEZE treatment.

Compound	**PMC-3-Cl**	**PMC-3-Br**	**PMC-3-I**
Radiation type,<br>Wavelength / Å	Mo Kα<br>0.71073	Mo Kα<br>0.71073	Mo Kα<br>0.71073
Empirical formula	$C_{72}H_{44}O_{16}N_{12}Cl_4Zn_3$	$C_{72}H_{44}O_{16}N_{12}Br_4Zn_3$	$C_{72}H_{44}O_{16}N_{12}I_4Zn_3$
Formula weight<br>/ g mol^{-1}	1663.04	1840.88	2028.84
Crystal system	Orthorhombic	Orthorhombic	Orthorhombic
Space group	*Cmme*	*Cmme*	*Cmme*
Crystal size / mm	0.265 × 0.115 × 0.030	0.251 × 0.092 × 0.031	0.331 × 0.060 × 0.025
Crystal color	Black	Black	Black
Crystal shape	Rectangular prism	Rectangular prism	Rectangular prism
Unit cell<br>Dimensions / Å	a = 31.9728(8)<br>b = 39.2757(10)<br>c = 9.5955(3)	a = 32.1408(8)<br>b = 39.3430(10)<br>c = 9.5935(3)	a = 32.3760(13)<br>b = 39.5596(15)<br>c = 9.5701(6)
Volume / Å^3	V = 12049.6(6)	V = 12131.1(6)	V = 12257.2(10)
Temperature / K	120	120	120
Z	4	4	4
Density<br>(calculated)<br>/ Mg m^{-3}	0.917	1.008	1.099
Absorption<br>coefficient / mm^{-1}	0.727	1.952	1.635
R_1, $wR_2[I > 2\sigma(I)]$	0.0713, 0.2575	0.1047, 0.3155	0.0881, 0.2683
R_1, wR_2[all data]	0.1066, 0.2750	0.1360, 0.3341	0.1064, 0.2821
F(000)	3352	3640	3928
Goodness of fit on<br>F^2	1.139	1.108	1.096

Stacking structure of PMC-3-Br

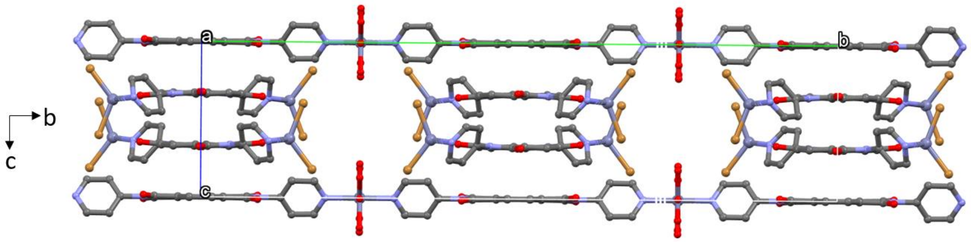

Figure S1. Side view of the stacking structure of **PMC-3-Br** viewed along the *a* axis.

Voids accommodating lattice solvent molecules in PMC-3

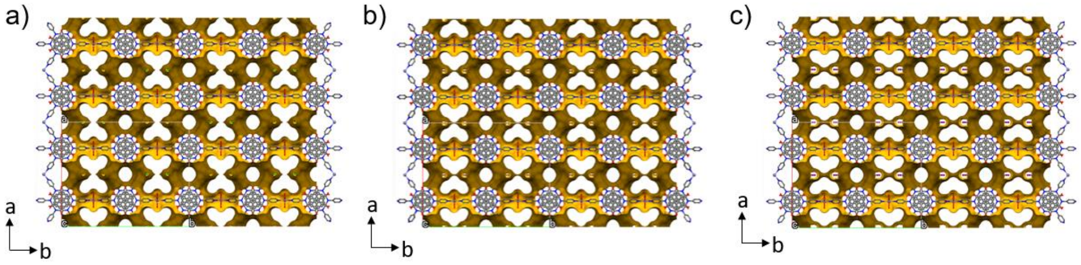

Figure S2. Crystal structure with the representation of voids in **PMC-3** for X = a) Cl, b) Br, and c) I. Solvent molecules are omitted for clarity. The contact surface of the voids (brown) is shown by applying a probe radius of 1.65 Å and approx. grid spacing of 0.2 Å. The void volumes are calculated as 6532.79 Å^3 (54.2%) for Cl, 6504.30 Å^3 (53.6%) for Br, and 6481.85 Å^3 (52.9%) for I.

¹H NMR spectra

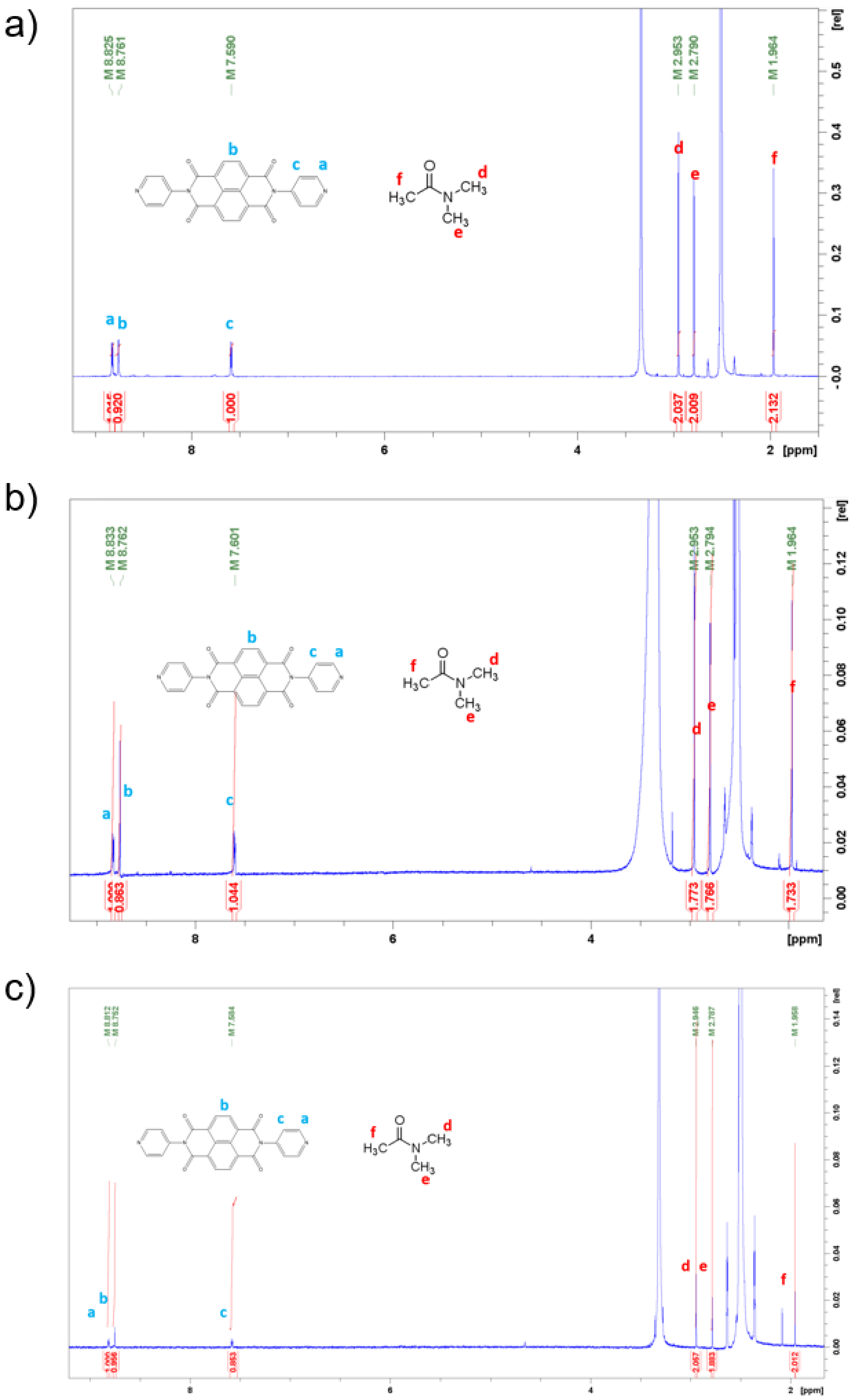

Figure S3. ^{1}H NMR spectra of a) **PMC-3-Cl**, b) **PMC-3-Br** and c) **PMC-3-I** dissolved in d_6-DMSO. The peaks at 3.32 and 2.51 ppm correspond to water and residual DMSO molecules in the NMR solvent.

Thermogravimetry analysis (TGA) of PMC-3

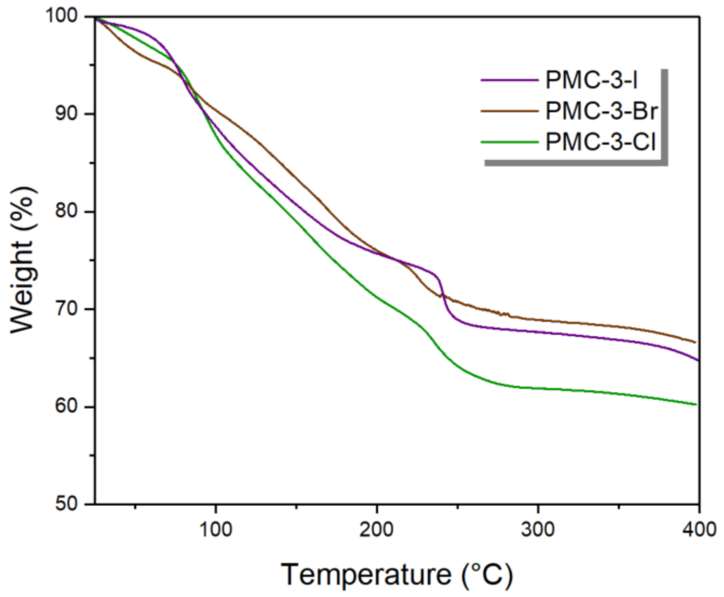

Figure. S4. Thermogravimetry analysis of **PMC-3** at a heating rate of 5 K min^{-1}.

PXRD patterns of PMC-3-Br after heating

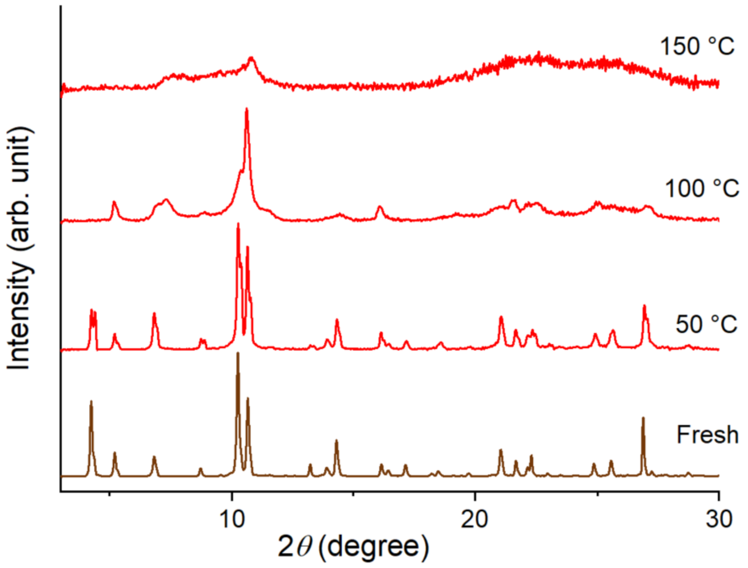

Figure S5. PXRD patterns of **PMC-3-Br** measured after heating to 50, 100, and 150 °C.

Gas sorption isotherm

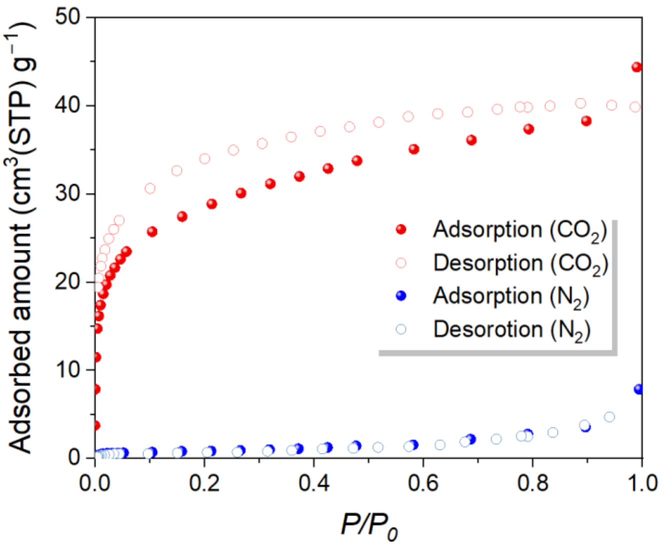

Figure S6. Gas sorption isotherms of **PMC-3-Br** for N_2 (blue) at 77 K and CO_2 (red) at 195 K.

Magnetic susceptibility

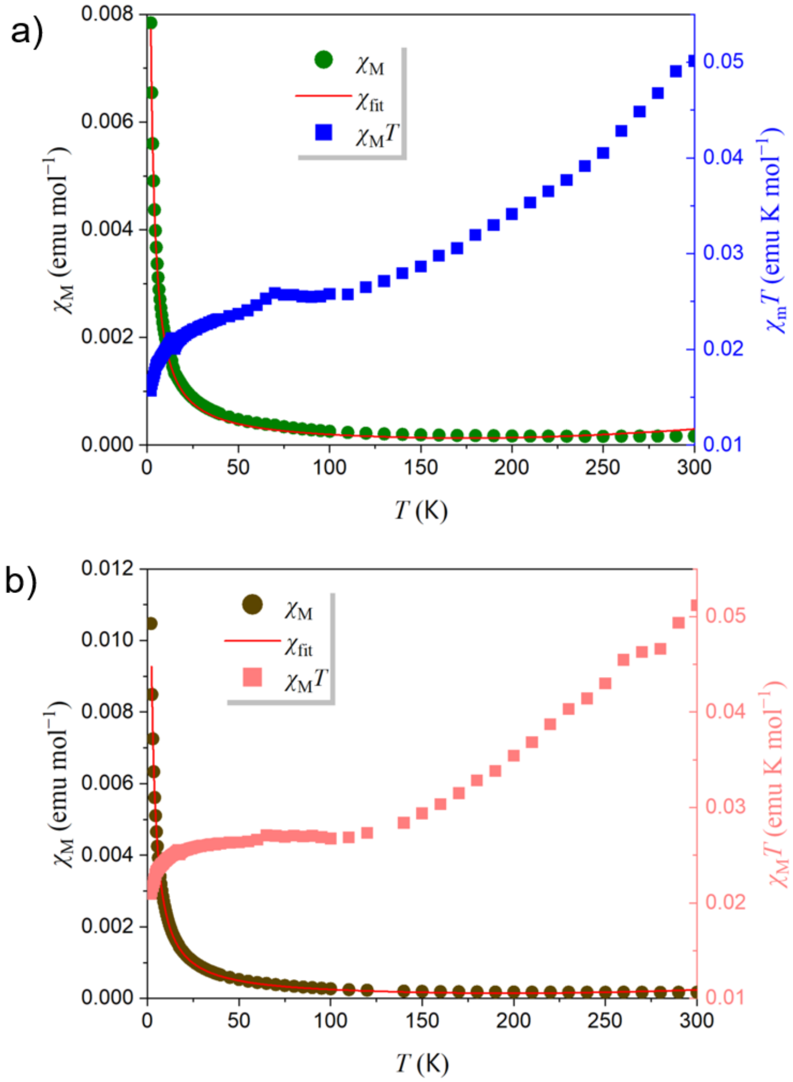

Figure S7. Temperature dependence of molar spin susceptibility (χ_M–T plot; circles, left vertical axis) and that of $\chi_M T$ values ($\chi_M T$–T plot; squares, right vertical axis) of a) **PMC-3-Cl** and b) **PMC-3-Br**. The red solid lines are the fitting line (χ_{fit}) by the model consisting of a Curie term and a singlet-triplet thermal excitation component.

The following equation and parameters are used for the fitting lines in Figure S7.

$$\chi_{fit} = \chi_0 + 2n \cdot \frac{N_A g^2 \cdot \frac{1}{2} \cdot \frac{3}{2} \cdot {\mu_B}^2}{3k(T-\theta)} + \frac{N_A g^2 {\mu_B}^2}{kT} \cdot \frac{2}{\exp\left(\frac{-E_A}{kT}\right) + 3}$$

χ_0: Temperature-independent paramagnetism
n: Spin density
N_A: Avogadro constant
g: g-value (2.0021 for **PMC-3-Cl** and 2.0015 for **PMC-3-Br**)

μ_B: Bohr magneton

k: Boltzmann constant

T: Temperature

E_A: Activation energy of singlet-triplet excitation

Table S2. Parameters used in fitting χ

	**PMC-3-Cl**	**PMC-3-Br**
χ_0 (emu mol^{-1})	1.1×10^{-6}	0
n	0.033	0.026
θ (K)	–0.56	–0.40
E_A/k (K)	–1330	–1450
Coefficient of determination (R^2)	0.99607	0.99931

Electronic band calculation

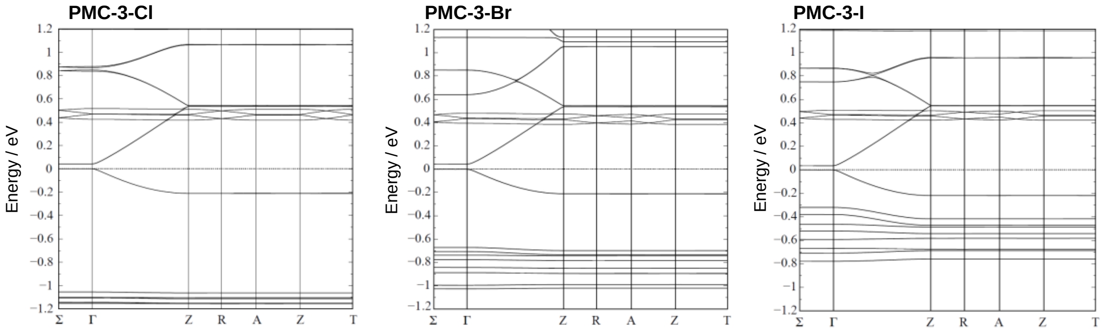

Figure S8. Electronic band structures of three **PMC-3** compounds calculated using first-principles methods with the GGA–PBE functional.

Electrical conductivities of single crystals

Table S3. Electrical conductivities of **PMC-3-Cl** from three single crystals measured at 300 K under N_2 atmosphere.

Batch	1	2	3
σ_{300K} (S cm^{-1})	1.6×10^{-3}	0.77×10^{-3}	1.6×10^{-3}
Activation energy E_a (meV)	125.1	138.1	135.0

Table S4. Electrical conductivities of **PMC-3-Br** from three single crystals measured at 300 K under N_2 atmosphere.

Batch	1	2	3
σ_{300K} (S cm^{-1})	1.8×10^{-3}	1.3×10^{-3}	5.5×10^{-3}
Activation energy E_a (meV)	154.6	126.0	169.6

Photographs of crystals as synthesized and after conductivity measurement

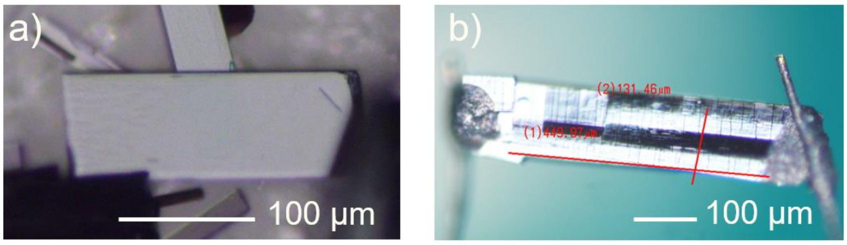

Figure S9. Photographs of **PMC-3-Br** crystals: a) in the fresh state and b) after single-crystal conductivity measurement. Numerous cracks formed in b) due to the liberation of the crystal solvent.

Electrical conductivities of pressed pellets

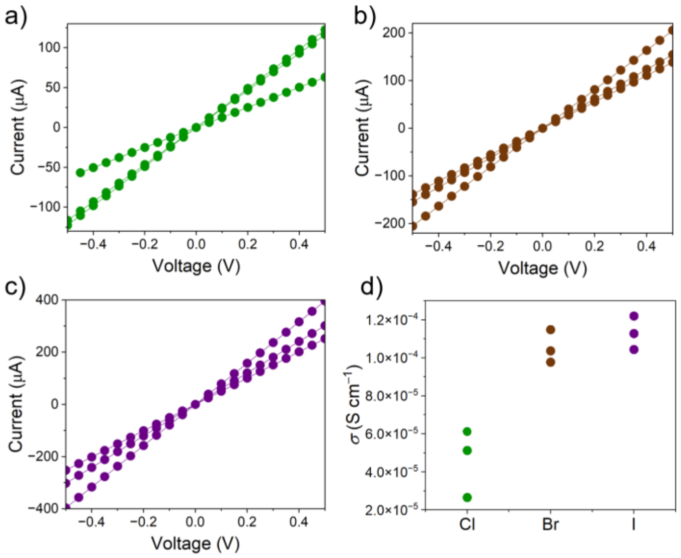

Figure S10. Current–voltage *(I–V)* characteristics of a) **PMC-3-Cl,** b) **PMC-3-Br**, and (c) **PMC-3-I** measured in pressed pellet form. d) Plot showing the distribution of calculated conductivity for **PMC-3** from three different batches.

Table S5. Electrical conductivities of **PMC-3** calculated from *I–V* plot measured in pressed-pellet form at room temperature.

σ_{RT} (S cm^{-1})	**PMC-3-Cl**	**PMC-3-Br**	**PMC-3-I**
Batch 1	5.1×10^{-5}	1.1×10^{-4}	1.1×10^{-4}
Batch 2	6.1×10^{-5}	1.0×10^{-4}	1.2×10^{-4}
Batch 3	2.7×10^{-5}	0.98×10^{-4}	1.0×10^{-4}
**Average**	$\mathbf{4.6 \times 10^{-5}}$	$\mathbf{1.0 \times 10^{-4}}$	$\mathbf{1.1 \times 10^{-4}}$

Calculated $\phi\Sigma\mu$ of PMC-3 from FP-TRMC measurement

Table S6. Calculated $\phi\Sigma\mu$ of **PMC-3**.

Sample	$\phi\Sigma\mu$ / cm^2 V^{-1} s^{-1}
**PMC-3-Cl**	1.3×10^{-4}
**PMC-3-Br**	1.7×10^{-4}
**PMC-3-I**	4.1×10^{-4}

Solid-state cyclic voltammogram of PMC-3-Br

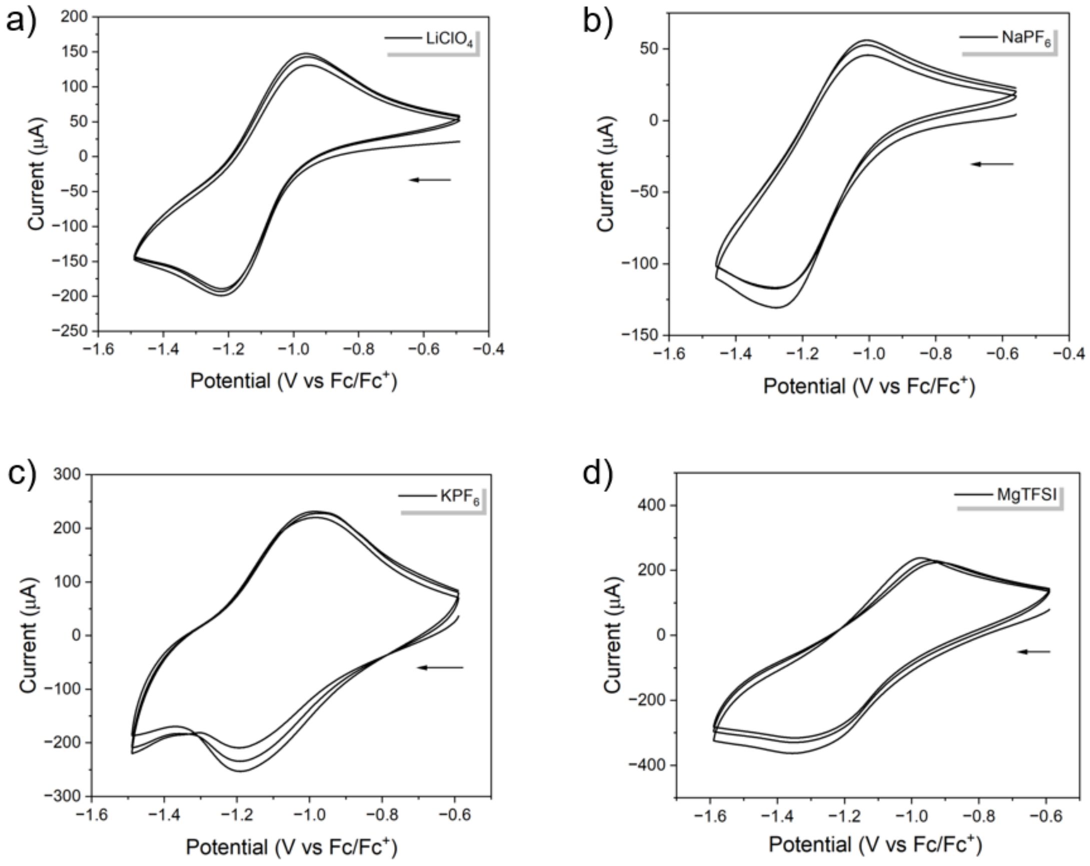

Figure S11. Solid-state cyclic voltammogram of **PMC-3-Br** recorded in 0.1 M (a) $LiClO_4$, (b) $NaPF_6$, (c)KPF_6 and MgTFSI in dry acetonitrile at a scan rate of 100 mVs^{-1} under a nitrogen flow. The arrows indicate the direction of the forward scan. The $E_{1/2}^{red}$ are calculated as −1.09 V, −1.14 V, −1.09 V, and −1.17 V for $LiClO_4$, $NaPF_6$, KPF_6 and MgTFSI, respectively.

Solid-state cyclic voltammogram of NDI-py

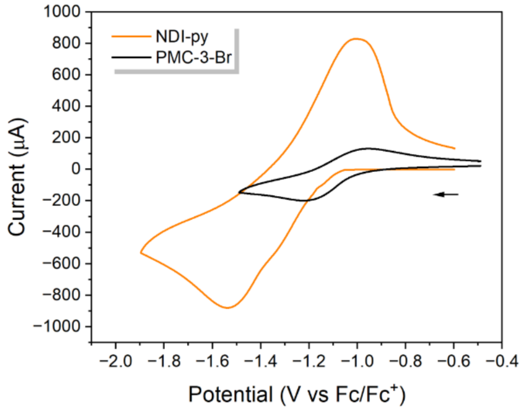

Figure S12. Solid-state cyclic voltammogram of NDI-py and **PMC-3-Br** recorded in 0.1 M $LiPF_6$ and $LiClO_4$, respectively, in dry acetonitrile at a scan rate of 100 mVs^{-1} under a nitrogen flow. The arrows indicate the direction of the forward scan. The $E_{1/2}^{red}$ are calculated to be −1.27 V and −1.09 V for NDI-py and **PMC-3-Br**, respectively.

Solid-state UV-vis spectroelectrochemistry (SEC) of PMC-3-Br

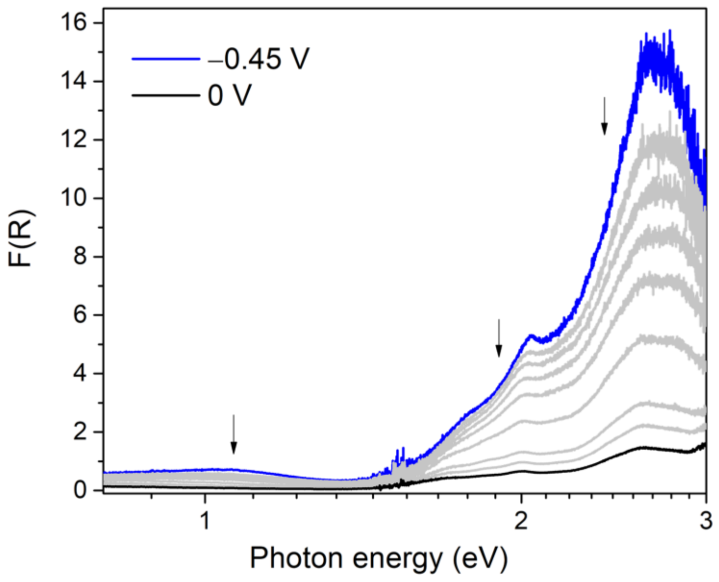

Figure S13. Solid-state SEC of **PMC-3-Br** in contact with 0.1 M $LiBF_4$ in MeCN. The applied potential was scanned from −0.45 V (blue) to 0 V (black). Gray lines show the spectral progression, and the arrows indicate the direction of this progression.

Solid-state EPR spectroelectrochemistry (SEC) of PMC-3-Br

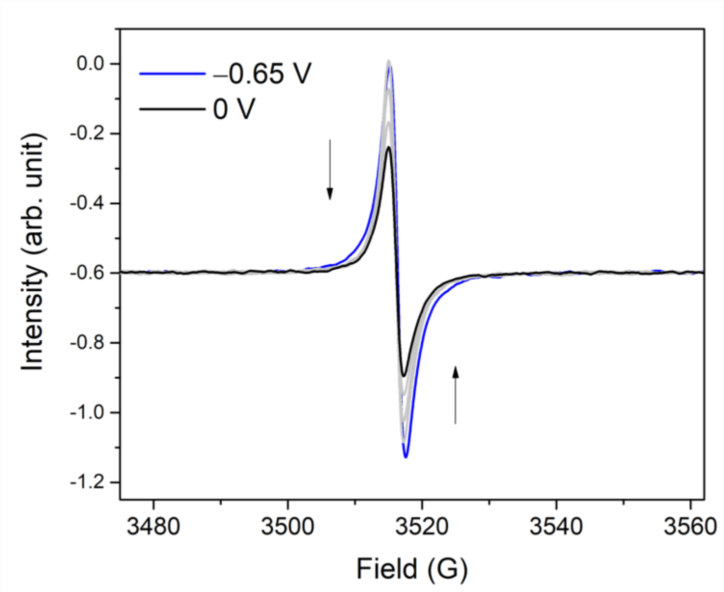

Figure S14. Solid-state EPR SEC of **PMC-3-Br** in contact with 0.1 M $LiBF_4$ in MeCN. The applied potential was scanned from −0.65 V (blue) to 0 V (black). Gray lines show the spectral progression and the arrows indicate the direction of this progression.

Reference

[1] L.-Z. Liao, X.-Y. Wu, J.-P. Yong, H.-L. Zhang, W.-B. Yang, R. Yu, C.-Z. Lu, Anion−π Interaction-Directed Assembly of Polyoxometalate-Based Host–Guest Compounds and Its Contribution to Photochromism, *Cryst. Growth. Des.* 2015, **15**, 4952–4958.

[2] G. M. Sheldrick, SHELXT–Integrated space-group and crystal structure determination, G. M. *Acta Cryst.* 2015, **A71**, 3–8.

[3] G. M. Sheldrick, Crystal structure refinement with SHELXL, *Acta Cryst.* 2015, **C71**, 3–8.

[4] O. V. Dolomanov, L. J. Bourhis, R. J. Gildea, J. A. K. Howard, H. Puschmann, OLEX2: a complete structure solution, refinement and analysis program, *J. Appl. Cryst.*, 2009, **42**, 339–341.

[5] A. L. Spek, PLATON SQUEEZE: a tool for the calculation of the disordered solvent contribution to the calculated structure factors, *Acta Cryst.* 2015, **C71**, 9–18.

[6] P. M. Usov, C. Fabian, D. M. D'Alessandro, Rapid determination of the optical and redox properties of a metal-organic framework via in situ solid state spectroelectrochemistry, *Chem. Commun.*, 2012, **48**, 3945–3947.

[7] B. Ding, B. Chan, N. Proschogo, M. B. Solomon, C. J. Kepert, D. M. D'Alessandro, A cofacial metal–organic framework based photocathode for carbon dioxide reduction, *Chem. Sci.*, 2021, **12**, 3608–3614.

[8] G. Kresse, J. Hafner, *Ab initio* molecular dynamics for liquid metals, *Phys. Rev. B* **1993**, *47*, 558.

[9] G. Kresse, J. Furthmüller, Efficiency of ab-initio total energy calculations for metals and semiconductors using a plane-wave basis set, *Comput. Mater. Sci.* **1996**, *6*, 15–50.

[10] G. Kresse, J. Furthmüller, Efficient iterative schemes for *ab initio* total-energy calculations using a plane-wave basis set, *Phys. Rev. B* **1996**, *54*, 11169.

[11] J. P. Perdew, K. Burke, M. Ernzerhof, Generalized Gradient Approximation Made Simple, *Phys. Rev. Lett.* **1996**, *77*, 3865–3868.
